# Health impact of E-cigarettes: a prospective 3.5-year study of regular daily users who have never smoked

**DOI:** 10.1038/s41598-017-14043-2

**Published:** 2017-11-17

**Authors:** Riccardo Polosa, Fabio Cibella, Pasquale Caponnetto, Marilena Maglia, Umberto Prosperini, Cristina Russo, Donald Tashkin

**Affiliations:** 1 Centro per la Prevenzione e Cura del Tabagismo (CPCT), Azienda Ospedaliero-Universitaria “Policlinico-V. Emanuele”, Università di Catania, Catania, Italy; 2 Institute of Internal and Emergency Medicine, Azienda Ospedaliero-Universitaria “Policlinico-V. Emanuele”, Università di Catania, Catania, Italy; 3 Dipartimento di Medicina Clinica e Sperimentale, University of Catania, Catania, Italy; 4 National Research Council of Italy, Institute of Biomedicine and Molecular Immunology, Palermo, Italy; 5 Ospedale “San Vincenzo” - ASP Messina, Taormina, ME Italy; 6 MCAU ARNAS Garibaldi, Catania, Italy; 7 David Geffen School of Medicine at the University of California, Los Angeles (UCLA), Los Angeles, California, USA

## Abstract

Although electronic cigarettes (ECs) are a much less harmful alternative to tobacco cigarettes, there is concern as to whether long-term ECs use may cause risks to human health. We report health outcomes (blood pressure, heart rate, body weight, lung function, respiratory symptoms, exhaled breath nitric oxide [eNO], exhaled carbon monoxide [eCO], and high-resolution computed tomography [HRCT] of the lungs) from a prospective 3.5-year observational study of a cohort of nine daily EC users (mean age 29.7 (±6.1) years) who have never smoked and a reference group of twelve never smokers. No significant changes could be detected over the observation period from baseline in the EC users or between EC users and control subjects in any of the health outcomes investigated. Moreover, no pathological findings could be identified on HRCT of the lungs and no respiratory symptoms were consistently reported in the EC user group. Although it cannot be excluded that some harm may occur at later stages, this study did not demonstrate any health concerns associated with long-term use of EC in relatively young users who did not also smoke tobacco.

## Introduction

Electronic cigarettes (ECs) are battery powered electronic devices. Puffing on an EC heats up an element (most commonly, a metal coil) that vaporizes a solution (e-liquid) mainly consisting of propylene glycol, vegetable glycerin, distilled water, and flavorings that may or may not contain liquid nicotine. The user inhales the aerosol generated by vaporizing the e-liquid in a process commonly referred to as “vaping”. ECs do not contain tobacco, do not create smoke and do not rely on combustion to operate. These consumer products have been rapidly gaining ground on conventional cigarettes among smokers due to the expectation of reducing/quitting smoking^1–4^, the perception of being a less harmful alternative to cigarettes^1–6^, competitive price^7–9^ and because they allow the smoker to continue having a “smoking experience without smoking”^9–11^.

Although vapour toxicology under normal condition of use is less problematic than tobacco smoke^12–14^ and e-vapour products are estimated to be less harmful than combustible cigarettes^15–17^, there is concern as to whether chronic exposure to their residual toxicological load may nevertheless carry a risk for lung health^18–20^. Therefore, investigating the health impact of long term EC use is warranted.

Considering that inhalation is the exposure mechanism for EC use, the respiratory system is the primary target of any potential harmful effects of constituents in ECs aerosol emissions. No deterioration in lung function, airway responses, and respiratory symptoms could be observed in a 1-year prospective RCT of “healthy” smokers who were invited to quit or reduce their tobacco consumption by switching to ECs^20,21^. Of note, FEF25–75% (a sensitive measure of obstruction in the more peripheral airways)^20^, nitric oxide (a non-invasive biomarker of airway inflammation in airways disease, as well as in studies of environmental and occupational exposure)^21^ and carbon monoxide (a commonly used indicator of smoking abstinence that also reflects airway inflammation) in the exhaled breath returned to within normal limits^21^, with similar degree of normalization occurring in quitters who stopped using ECs as well as in quitters who were still using ECs. Overall, these preliminary studies do not appear to suggest negative respiratory health outcomes in smokers who have switched to ECs.

Nonetheless, very little is known about the long-term health effects of vaping. When investigating these health effects, it is important to consider that it is difficult (if not impossible) to disentangle responses driven by chronic exposure to EC aerosol emissions from those related to previous smoking history, unless one were to conduct studies on regular EC users who have never smoked. If EC aerosol emissions are much less harmful than tobacco smoke^15–17^, it can be hypothesized that long term vaping is less likely to cause significant harm to the respiratory system of regular daily EC users with no previous smoking history. This has never been formally tested in a research study.

Thus, aim of the study was to compare health outcomes between a cohort of daily EC users who have never smoked and a sample of never smokers and non-users of EC over a period of 3.5 years. Here we report findings from a prospective 3.5-year observational study comparing changes in health outcomes between a cohort of daily EC users who have never smoked and a reference group of never smokers and non-users of EC. Health outcomes included blood pressure (BP), heart rate (HR), body weight, lung function, respiratory symptoms, as well as exhaled biomarkers of airway inflammation (exhaled breath nitric oxide [eNO] and carbon monoxide [eCO]). EC users were also offered high-resolution computed tomography (HRCT) of the lungs at the end of follow-up to assess their risk for early signs of lung damage. This is the first study that has explored health effects of prolonged exposure to EC use in never smokers.

## Methods

### Participants and Study Design

Adult EC users (≥18 years old) were identified amongst a pool of regular Vape Shops customers. Vape shop owners who helped in a previous study^22^ were instructed to ask their regular clients a few questions about smoking history and EC use patterns. Customers who had never smoked or who reported having smoked less than 100 cigarettes in their lifetime were defined as never smokers^23^ and considered for inclusion. They also had to be daily EC users of ≥3 months. They were invited for a free medical check-up at the Centro per la Prevenzione e Cura del Tabagismo (CPCT) of the University of Catania. Age- and sex-matched non-smoking controls (and not using ECs) were selected from hospital staff and included as a reference (control) group. Subjects were recruited from June 2013 to September 2013 and data collection completed in March 2017. Participants came to the CPCT in the mornings for their check-up visits during which vital signs (blood pressure - BP, heart rate - HR, body weight) as well as measurements of lung function, respiratory symptoms, and airway inflammation (eNO and eCO levels) were recorded. Details of EC-related products purchased (i.e. ECs hardware, e-liquid nicotine strengths and flavours) were also noted. Three additional follow-up visits were scheduled yearly for up to 3.5 years; follow-up visits 1 (F/up1), 2 (F/up2) and 3 (F/up3) were carried out at 12 (±1), 24 (±2) and 42 (±2) months after baseline visits, respectively. At F/up3, EC users were offered the additional option of undergoing lung HRCT. All these tests and measurements were carried out in accordance with relevant guidelines and regulations. University of Catania Ethics Review Board approved the study protocol and subjects gave informed consent prior to participation.

### BP, HR, and Body Weight

After a 5-minute rest, BP and HR measurements were obtained by a semi-automated oscillometric sphygmomanometer (Smart Pressure, CA-MI Snc, Parma, Italy). Two measurements in the sitting position, spaced 1–2 min apart, were obtained at each visit. Measurements were taken in the morning, and participants were instructed not to vape or consume caffeinated drinks for at least 60 min prior to each visit. The average of two measurements was considered for analysis. Participants removed shoes and heavy clothing and were weighed at each visit by using a mechanical scale (Seca, Intermed Srl, San Giuliano Milanese, Italia).

### Spirometry Procedure

A technician who was blinded to participants’ characteristics conducted spirometric tests according to American Thoracic Society/European Respiratory Society guidelines^24^. Prediction values for spirometric indices were the 2012 multi-ethnic reference values for spirometry for the 3–95-yr age range by Quanjer *et al*.^25^. Forced expiratory volume in one-second (FEV_1_), forced vital capacity (FVC), and maximum mid-expiratory flow (FEF_25–75%_) were obtained using a PC-based electronic spirometer (Micro Medical Spiro USB ML2525 with Spida 5 Software; CareFusion, Sesto Fiorentino, Firenze, Italy). At least three forced expiratory maneuvers spaced 1–2 min apart were obtained with subjects sitting comfortably. Measurements were taken in the morning and participants were instructed not to vape for at least 60 min prior to each visit. The best FVC and FEV_1_ were retained and FEF_25–75%_ was selected from the maneuver with the largest sum of FEV_1_ and FVC. For each subject, FEV_1_/FVC was also computed. A respiratory physician experienced in pulmonary function testing (RP) reviewed spirometry results for quality control. Only technically acceptable tests were used for data analyses. Individual spirograms are “acceptable” if they are free from artefacts, they have good starts, they show satisfactory exhalation, as per well-defined criteria^24^.

### Respiratory symptoms

Self-reported respiratory symptoms were verified at baseline and at each study follow-up visits by asking 4 yes/no questions:

1. For cough: “*Have you had any cough in the previous 2-weeks*?”
2. For wheeze: “*Have you heard any wheeze when breathing*?”
3. For shortness of breath: “*Have you been short of breath in the previous 2-weeks*?”
4. For tight chest: “*Have you had difficulty in breathing like a sensation of pressure on your chest*?”

### FeNO measurements

Exhaled nitric oxide measurements (in ppb) were performed according to the American Thoracic Society/European Respiratory Society guidelines^26^ using a hand-held FeNO meter (NIOX Mino, Aerocrine AB, Sweden). Expiratory manoeuvres were performed in the morning with participants sitting comfortably. Participants were instructed not to vape for at least 60 min prior to each visit. Only technically acceptable tests were used for data analyses.

### eCO measurements

Measurements (in ppm) were obtained from a single expiratory breath by using a hand-held eCO meter (Micro CO, Micro Medical Ltd, UK) according to the manufacturer’s recommendations. Expiratory manoeuvres were taken in the morning with participants sitting comfortably. Participants were instructed not to vape for at least 60 min prior to each visit.

### Lung HRCT

HRCT scans were obtained with Toshiba Aquilion (Toshiba Medical Systems, Tokyo, Japan). A high-resolution algorithm was applied and 1-mm axial slices at 10-mm intervals from lung apices to bases were reconstructed. Subjects were scanned during suspended end-inspiration in the prone position without intravenous contrast material. An experienced radiologist who was blinded to participants’ characteristics evaluated the HRCT scans for presence or absence of pathologic signs.

### Statistical Analyses

With a power of 80% and a type-I error (alpha) of 0.05 (5%), 7 subjects per group (14 in all) would be sufficient for detecting a change of 12% in FEV_1_ (i.e., the minimal clinically important difference for FEV_1_ according to ATS/ERS criteria). Our analysis, based on mean and SD of FEV_1_ in Control (N = 12) and Pure Vapers (N = 9) groups, for a type-I error (alpha) of 0.05 (5%), produced a power of 6.7%.

Only data from subjects completing all four study visits were included in the analyses. Parametric data were expressed as mean (±standard deviation [SD]) while non-parametric data expressed as median (and interquartile range [IQR]). Possible between-group differences at baseline were evaluated using one-way Analysis of Variance and Mann-Whitney U-test for normally and not normally distributed continuous variables, respectively. Differences in frequency distribution of categorical variables were evaluated by χ^2^ test. A Repeated Measures ANOVA model was used for evaluating changes in health effects indicators at different time points (4 time points: baseline, F/up 1, F/up 2, and F/up 3): health effects indicators were entered into the model as within factor for assessing changes with time, while the study group (EC users/Controls) was entered as between factor for evaluating its effect on possible changes. A p value of less than 0.05 was considered to indicate statistical significance. All analyses were performed with the Statistical Package for Social Science (SPSS for Windows version 20.0, Chicago, IL, USA).

## Results

### Participant Characteristics

A total of 16 [M 11; F 5; mean (±SD) age of 29.7 (±6.1) years] consecutive regular daily EC users of ≥3 months who had never smoked and 15 age- and sex-matched [M 10; F 5; mean (±SD) age of 32.5 (±7.0) years] non-smoking controls (and not using ECs) consented to participate and were included in the study. From the EC users, four were lost to follow-up (no shows) and three were excluded, as they did not comply with inclusion criteria on review (two stopped vaping and one began vaping only sporadically). From the reference group, one was lost to follow-up (the subject moved to another city) and two were excluded, as they did not comply with inclusion criteria on review (both started tobacco smoking). Complete datasets were available from 9 EC users and 12 control subjects; their characteristics at baseline were not significantly different (with the exception of HR) and are presented in Table 1.

**Table 1:** Baseline characteristics of the study sample presented separately for EC users and control subjects who completed all four study visits.

	EC users (n = 9)	Control subjects (n = 12)	p value
Sex (M/F)	6/3	8/4	—^†^
Age (yrs, mean ± SD)	26.6 ± 6.0	27.8 ± 5.2	0.61^‡^
Duration of vaping hx (months, median [range])	8 (3.5–15)	N/A	—
Daily e-liquid consumption (ml, median [range])	4.0 (2–5)	N/A	—
E-liquid nicotine strengths (%)	0%: 3	N/A	
	0.9%: 2		
	1.2%: 2		
	1.6%: 1		
	1.8%: 1		
E-liquid flavour type	Tobacco: 7	N/A	
	Mint: 1		
	Fruit: 1		
Device type	Advanced refillable*: 4	N/A	
	Standard refillable**: 5		
Weight (kg, mean ± SD)	71.3 ± 11.2	72.9 ± 11.8	0.76^‡^
Systolic blood pressure (mmHg, mean ± SD)	115 ± 9	117 ± 9	0.59^‡^
Diastolic blood pressure (mmHg, mean ± SD)	79 ± 6	74 ± 9	0.20^‡^
Heart rate (beats/min, mean ± SD)	72 ± 7	79 ± 9	0.04^‡^
eCO (ppm, median and IQ range)	5.0 (3.5–7.3)	4.0 (3.5–7.5)	0.97^¥^
FeNO (ppb, median and IQ range)	21.1 (16.2–24.5)	18.6 (17.6–25.7)	0.75^¥^
FEV_1_ (% predicted, mean ± SD)	95.9 ± 9.4	104.8 ± 11.3	0.09^‡^
FVC (% predicted, mean ± SD)	102.0 ± 8.3	108.0 ± 8.4	0.12^‡^
FEV_1_/FVC (%, mean ± SD)	78.5 ± 3.5	81.4 ± 5.0	0.07^‡^
FEF_25–75%_ (% predicted, mean ± SD)	75.3 ± 9.2	82.6 ± 18.7	0.30^‡^

^†^χ^2^ test.

^‡^one-way Analysis of Variance.

^¥^Mann-Whitney U-test.

^*^Advanced refillable device included: Provari, Innokin, Joyetech eVIC, Avatar Puff.

^**^ *Standard refillable device: assorted EGO style products*.

Six of the nine EC users were consuming nicotine-containing e-liquid at baseline as well as by the end of study, although at a lower strength. Three have been consuming zero-nicotine strength e-liquid throughout consistently the 3.5 years follow-up. Also consistent over time was the consumption of preferred flavours (i.e. tobacco flavours). Some participants switched from standard refillables (i.e. assorted EGO style products) to more advanced refillable devices (including Provari, Innokin, Joyetech eVIC, Avatar Puff*)*.

### BP, HR, and Body Weight

Changes in systolic BP, diastolic BP, HR and body weight from baseline and between study groups are shown in Table 2. No significant changes from baseline were observed at any follow-up study visits in the EC group. No significant difference was found between EC users and control subjects. Because of the small sample size, we checked all individual datasets one by one to detect signs of negative changes and found no such changes (even among EC users consuming nicotine-containing e-liquids).

**Table 2:** Changes in health effect indicators at each follow-up visit, separately for EC users and control subjects who completed all four study visits.

		Baseline	F/up 1	F/up 2	F/up 3	Between effect p value
FEV_1_ (l, mean ± SD)	EC users	3.82 ± 0.78	3.81 ± 0.78	3.78 ± 0.71	3.87 ± 0.76	0.30
	Control subjects	4.08 ± 0.30	4.06 ± 0.28	4.03 ± 0.26	4.11 ± 0.30	
FVC (l, mean ± SD)	EC users	4.93 ± 0.95	4.80 ± 0.82	4.82 ± 0.91	4.87 ± 0.83	0.61
	Control subjects	5.03 ± 0.48	4.97 ± 0.42	5.01 ± 0.45	5.02 ± 0.42	
FEV_1_/FVC (%, mean ± SD)	EC users	78.49 ± 3.46	79.01 ± 3.63	78.46 ± 2.34	79.08 ± 2.83	0.09
	Control subjects	81.45 ± 5.03	82.02 ± 4.67	80.86 ± 6.18	82.06 ± 4.25	
FEF_25–75%_ (l/min, mean ± SD)	EC users	3.29 ± 0.70	3.29 ± 0.60	3.30 ± 0.75	3.33 ± 0.64	0.36
	Control subjects	3.43 ± 0.64	3.49 ± 0.61	3.53 ± 0.57	3.56 ± 0.58	
Weight (kg, mean ± SD)	EC users	71.3 ± 11.2	72.9 ± 11.5	73.3 ± 11.4	72.2 ± 11.2	0.95
	Control subjects	72.9 ± 11.8	74.0 ± 12.1	73.2 ± 12.3	73.6 ± 11.8	
Systolic blood pressure (mmHg, mean ± SD)	EC users	115 ± 9	116 ± 5	114 ± 9	118 ± 10	0.82
	Control subjects	117 ± 9	117 ± 10	116 ± 10	116 ± 9	
Diastolic blood pressure (mmHg, mean ± SD)	EC users	79 ± 6	78 ± 4	73 ± 9	76 ± 8	0.50
	Control subjects	74 ± 9	76 ± 6	75 ± 9	73 ± 9	
Heart rate (beats/min, mean ± SD)	EC users	72 ± 7	71 ± 9	71 ± 9	71 ± 7	0.15
	Control subjects	79 ± 9	78 ± 8	76 ± 8	78 ± 9	
eCO (ppm, median and IQ range)	EC users	5.0 [3.5–7.3]	4.0 [2.8–6.0]	3.0 [3.0–5.8]	4.0 [2.8–6.3]	0.21
	Control subjects	4.0 [3.5–7.5]	5.5 [4.0–6.5]	7.0 [3.5–8.0]	5.0 [5.5–6.0]	
FeNO (ppb, median and IQ range)	EC users	21.1 [16.2–24.5]	19.7 [17.2–22.3]	18.9 [18.2–24.7]	20.0 [18.2–22.7]	0.89
	Control subjects	18.6 [17.6–25.7]	19.4 [16.0–25.1]	18.7 [16.9–22.0]	20.0 [16.2–23.4]	

Within effect was time, between effect was belonging to EC user group or Control subjects group.

### Lung Function

**Figure 1:**
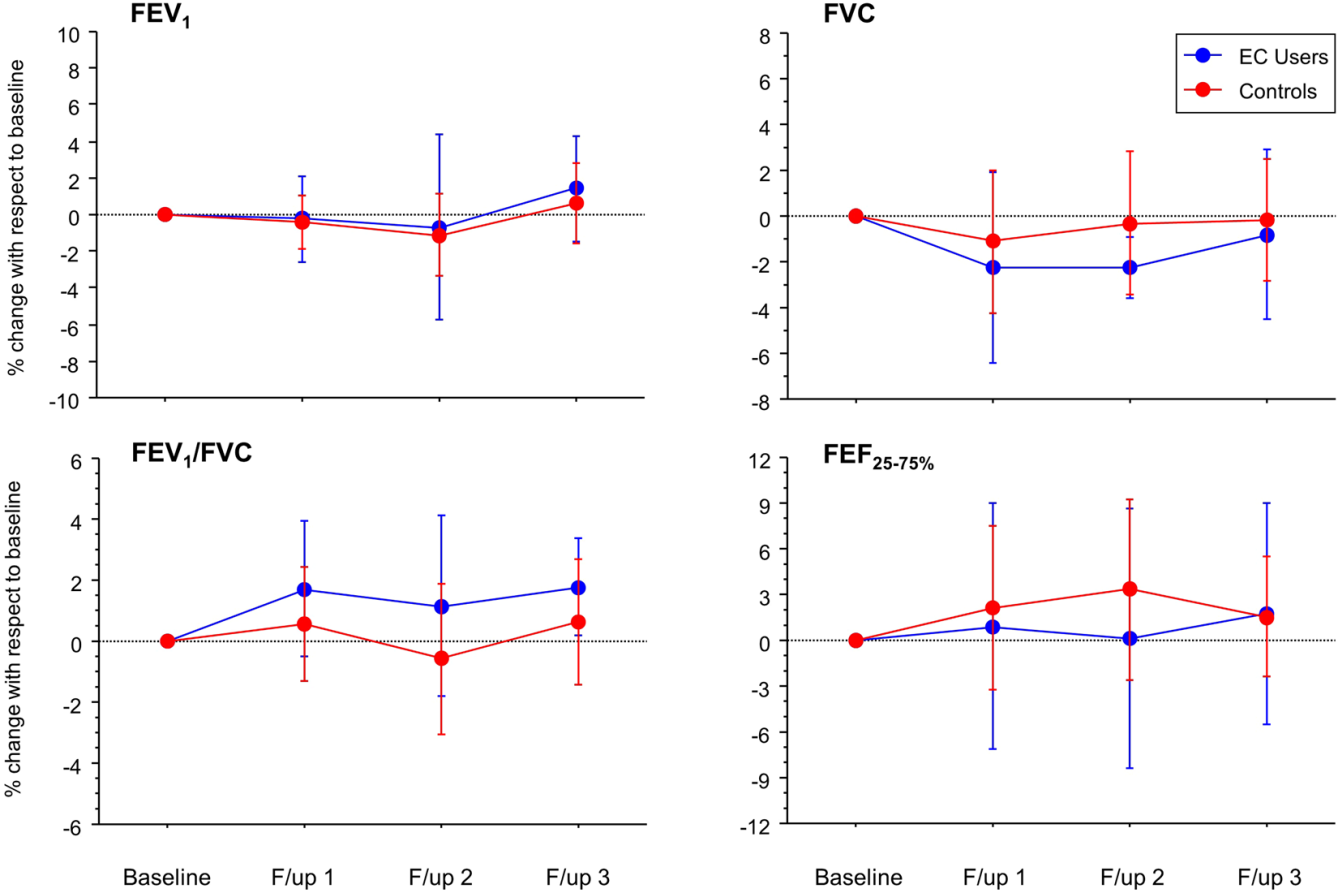
(**A**–**D**) Time trends (means ± SD) of FEV1 (panel A), FVC (panel B), FEV1/FVC (panel C) and FEF25–75 (panel D) at baseline (BL), and at follow-up visits at year-1 (F/up 1), year-2 (F/up 2) and year 3,5 (F/up 3), separately for daily EC users who have never smoked (blu lines) and controls (red lines).

Changes in FEV1, FVC, %FEV1/FVC and FEF25–75 from baseline and between study groups are shown in Table 2 and Fig. 1. No significant change from baseline was observed over the 3.5-years observation period in the EC group (Fig. 1, panels A–D). No significant difference was found between EC users and control subjects. None of the lung function variables showed a significant between-group (i.e., EC users/Controls) effect (Table 2; Fig. 1, panels A–D). Again, because of the small sample size, we checked all individual datasets one by one to detect signs of negative changes and found no such changes, even among those with the highest e-liquid consumption (5 ml/day) and longest vaping hx (57 months).

### Respiratory Symptoms

None of the participants in this study reported any wheezing, shortness of breath, or chest tightness. Cough was reported by one EC user at baseline and by another at F/up2. In the control group, three participants reported cough on three separate occasions. Of note, study participants reported no severe adverse reactions.

### FeNO and eCO measurements

**Figure 2:**
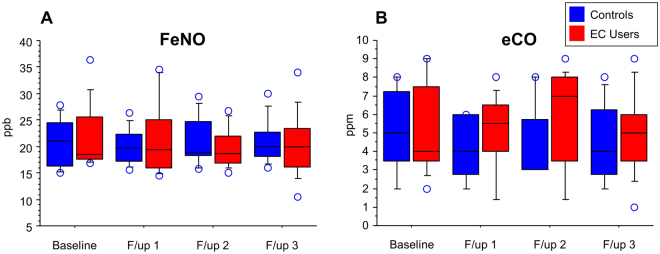
(**A**–**B**) Box-plot representation of time trends of exhaled nitric oxide (panel A) and exhaled carbon monoxide (panel B) at baseline (BL), and at follow-up visits at year-1 (F/up 1), year-2 (F/up 2) and year 3,5 (F/up 3), separately for daily EC users who have never smoked (red boxes) and controls (blu boxes). Median values are shown as horizontal bars.

Changes in FeNo and eCO from baseline and between study groups are shown in Table 2. No significant change from baseline was observed over the 3.5-years observation period in the EC group (Fig. 2, panels A,B). No significant difference was found between EC users and control subjects. Repeated Measures ANOVA showed that no between-group (i.e., EC users/Controls) effect in either FeNO or eCO (Table 2; Fig. 2, panels A,B). As with the other outcomes note above, we checked all individual datasets one by one to detect signs of negative changes and found no such changes, even among those with the highest e-liquid consumption and longest vaping hx”.

### Lung HRCT

HRCT scans were obtained in 8/9 EC users (one had claustrophobia and refused undergoing scanning). Visual assessment of the HRCT scans showed no pathological findings. In particular, no CT features compatible with early signs of COPD (i.e. parenchymal micronodules, ground-glass opacity, or macroscopic emphysema) or lipoid pneumonia or popcorn lung disease were present.

Of note, no early pathological signs were observed in subjects with the highest e-liquid consumption (i.e. 5 mls/day) and longest overall vaping hx (i.e. 57 months).

## Discussion

This small study, the first of its kind to date, found no detectable changes in lung health in never smokers who have been regularly vaping for at least 4 years. Daily exposure to ECs aerosol emissions caused no significant changes in any of the health outcomes investigated, including measures of lung function and lung inflammation. Moreover, no significant structural abnormalities could be identified on HRCT of the lungs and no respiratory symptoms were consistently reported. In spite of the small sample size and lack of comparison to smokers, careful examination of long-term health effects of EC use in a rare cohort of regular daily users who have never smoked in their life may contribute to the current understanding of the potential health risks associated with EC use.

Six of the nine EC users who completed the study were still consuming nicotine-containing e-liquids as reported on their last visit. Tobacco combustion products, not nicotine, cause most of the adverse health effects of smoking^27^. However, there is concern that some adverse cardiovascular effects may be related to nicotine per se due to its ability to cause hemodynamic changes (increase in heart rate, a transient rise in blood pressure, vasoconstriction of coronary and other vascular beds), adverse effects on lipids and induction of insulin resistance^27^. In this study, no significant changes in systolic BP, diastolic BP or HR were observed in the EC user group throughout the study. Moreover, no notable individual changes were observed in any of the vapers consuming nicotine-containing e-liquids. Thus, consumption of low dose nicotine did not seem to have significant adverse cardiovascular effects, as shown in recent EC studies of healthy smokers^28^ and smokers with arterial hypertension^29^. Also, the latest US Surgeon General’s report that examined harm from tobacco and nicotine has concluded that - although it may adversely affect foetus and adolescent brain development – nicotine does not contribute to smoking-related diseases^30^.

Given that particle size in ECs aerosols is well within the respiratory range^31,32^, these particles can penetrate deeply within the lungs; therefore the concern that long-term exposure to ECs aerosol emissions may carry some health risk is reasonable^18,19,33^. However, in this study no significant changes in lung function, respiratory symptoms, FeNO or eCO measurements were found. Furthermore, no noticeable individual changes were observed in any of the vapers including those with the most significant exposure history. Some acute EC trials in healthy smokers have reported transient changes in respiratory effects^34^, but others have not confirmed these observations^35,36^. Long-term studies in healthy smokers^20,21^ and smokers with asthma and COPD^37,38^ switching to EC use have shown not only no clinically significant adverse respiratory effects, but, to the contrary, a mitigation of the harmful effects of smoked tobacco on the lung.

In addition, substantial improvement in respiratory symptoms has been reported in a large internet survey of 19.000 smokers who switched to vaping^1^. In those diagnosed with asthma (n = 1173) or COPD (n = 1062) improvement in respiratory symptoms after switching was reported in 65.4% and in 75.7% of the respondents, respectively. Worsening after switching was reported only in 1.1% of the asthmatics and 0.8% of the respondents with COPD.

Lung function tests and self-reported respiratory symptoms may not be sensitive enough to detect early potential pathologic changes that may occur in response to chronic inhalation of EC aerosol emissions. HRCT can be used to better identify distribution and extent of early evidence of lung damage^39^. Early signs of lung damage, such as parenchymal micronodules, ground-glass attenuation and emphysematous changes, have been described in asymptomatic smokers with and without spirometric abnormalities^40–42^. However, in this study no CT features indicative of early signs of lung damage were present in any of the EC users.

Flavorings in the e-liquid are generally considered safe to eat, but have largely unknown effects on the lung when heated and inhaled. Chronic exposure to high levels of diacetyl - a flavoring substance commonly used in the food industry for its appealing buttery aroma - in microwave popcorn workers has been shown to be associated with cases of bronchiolitis obliterans (i.e. “popcorn lung”)^43,44^. Although many vaping liquids may contain high concentrations of diacetyl^45,46^, there is no report that this has caused bronchiolitis obliterans in EC users. In this study, no features consistent with early sign of bronchiolitis obliterans were described in any of the EC users undergoing HRCT.

In the present study, over a period of about 4 years, none of the EC users started smoking tobacco cigarettes (two stopped vaping completely) and two never smokers from the reference group (never smokers, not using ECs) started smoking. Whether the use of ECs is a gateway to (or out of) smoking remains matter of debate^47^.

Some of the strengths of this study include the relatively long follow up period, the detailed vaping history, careful characterization of the study participants and the use of a panel of different clinical, functional and inflammatory measures. But it has also some notable limitations.

In relation to health effects reporting, it is important to acknowledge that reasons for loss to follow up may include health problems. In our study, four EC users were lost to follow-up (no shows); these individuals may have quit ECs because they experienced negative health effects associated with their use. Hence, the risk for selection bias cannot be excluded.

The very small sample size minimizes the power to show both prevalent abnormalities at baseline and statistically significant changes from baseline over time. Nonetheless, it must be recognized that vapers who have never smoked are a very uncommon sub-population of ECs users; the 2014 Eurobarometer survey found about 0.1% of daily EC use in never smokers^48^. Hence, we were fortunate enough to recruit into the study such a carefully selected rare population of great importance to address the potential absolute risk of long term exposure to EC aerosol emissions disentangled from the effects of concomitant or former tobacco cigarette smoking. However, there is evidence of significant spirometry changes relatively early after initiation of smoking, even when analysing very small samples - as low as 13 smokers^49^. In any case, careful examination of the individual data on a case-by-case basis revealed no impairment in the health measures evaluated in any of the EC users in the study.

Another limitation is that the sample of relatively young subjects studied (mean age 27–28 years), who had had a generally short duration of regular EC use prior to entering the study (on average 8 months) and vaporized, on average, only a modest amount of e-liquid (about 4 ml/die), may not be representative of the general population of EC users who never smoked. Consequently, firm conclusions cannot be drawn from the results and additional studies in a larger and more diverse group of EC users are needed. On the other hand, vapers who have never smoked are likely to be relatively young^1,50^.

A related weakness is that since the age of our subjects was, on average, only in the mid-twenties, the normal age-related decline in lung function may not have yet commenced^51^, thus making it more difficult to show accelerated declines in response to any EC-related lung injury. Also, since duration and intensity of smoking are significant predictors of lung function decline among regular cigarette smokers^52^, the generally short duration and small amount of EC use by the vapers whom we studied may not have been sufficient to result in detectable lung damage. In any case, these deficiencies may guide other researchers to improve the design of similar studies.

Although no deterioration in lung health was detected during the 3.5 years follow-up, one could argue that no significant changes would have been detected among young healthy smokers during such a period as well. Comparison with a reference group of young smokers would have helped the interpretation of the results in EC users, thus making problematic to establish whether ECs are harmless or even less harmful than conventional cigarettes. Data documenting the effects of smoking on the lung over the first few years following initiation of smoking are limited. Tashkin and coll^49^ found that, over the five years between two visits at which spirometry was performed, those who initiated the smoking habit sometime during these 5 years (possibly, 2.5 years on average) had a relatively greater “negative” change in spirometric indices compared to those who never initiated the smoking habit. Niewoehner and coll^52^ found pathologic changes of the small airways at autopsy in young cigarette smokers who had died accidentally in motor vehicle accidents, indicating evidence of the harmful effects of smoking on the lung relatively early after initiation of smoking.

Another shortcoming is that HRCT scans were performed only at TLC, so that air-trapping (a sensitive measure of early lung damage in smokers with normal spirometry) could not be assessed^53^.

## Summary and Conclusions

In a small sample of young-adult never-smoking, daily EC users who were carefully followed for approximately 3½ years, we found no decrements in spirometric indices, development of respiratory symptoms, changes in markers of lung inflammation in exhaled air or findings of early lung damage on HRCT, when compared with a carefully matched group of never-smoking non-EC users. Even the heaviest EC users failed to exhibit any evidence of emerging lung injury as reflected in these physiologic, clinical or inflammatory measures. Moreover, no changes were noted in blood pressure or heart rate. Since the EC users who we studied were never smokers, potential confounding by inhalation of combustion products of tobacco were obviated.

While the sample size was small, the results of this study may provide some preliminary evidence that long-term use of ECs is unlikely to raise significant health concerns in relatively young users. Further studies in a larger sample of EC users with and without a history of tobacco smoking are warranted.

## Electronic supplementary material

Dataset 1
